# Comparison of the Manual, Semiautomatic, and Automatic Selection and Leveling of Hot Spots in Whole Slide Images for Ki-67 Quantification in Meningiomas

**DOI:** 10.1155/2015/498746

**Published:** 2015-07-09

**Authors:** Zaneta Swiderska, Anna Korzynska, Tomasz Markiewicz, Malgorzata Lorent, Jakub Zak, Anna Wesolowska, Lukasz Roszkowiak, Janina Slodkowska, Bartlomiej Grala

**Affiliations:** ^1^Warsaw University of Technology, Pl. Politechniki 1, 00-661 Warsaw, Poland; ^2^Nalecz Institute of Biocybernetics and Biomedical Engineering PAS, Trojdena 4, 02-109 Warsaw, Poland; ^3^Military Institute of Medicine, Szaserow 128, 04-141 Warsaw, Poland

## Abstract

*Background*. This paper presents the study concerning hot-spot selection in the assessment of whole slide images of tissue sections collected from meningioma patients. The samples were immunohistochemically stained to determine the Ki-67/MIB-1 proliferation index used for prognosis and treatment planning. *Objective*. The observer performance was examined by comparing results of the proposed method of automatic hot-spot selection in whole slide images, results of traditional scoring under a microscope, and results of a pathologist's manual hot-spot selection. *Methods*. The results of scoring the Ki-67 index using optical scoring under a microscope, software for Ki-67 index quantification based on hot spots selected by two pathologists (resp., once and three times), and the same software but on hot spots selected by proposed automatic methods were compared using Kendall's tau-b statistics. *Results*. Results show intra- and interobserver agreement. The agreement between Ki-67 scoring with manual and automatic hot-spot selection is high, while agreement between Ki-67 index scoring results in whole slide images and traditional microscopic examination is lower. *Conclusions*. The agreement observed for the three scoring methods shows that automation of area selection is an effective tool in supporting physicians and in increasing the reliability of Ki-67 scoring in meningioma.

## 1. Introduction

Immunohistochemistry (IHC) has become an important technique to both diagnostic pathology and clinical research, as it can help in the process of diagnosis, prognosis, and grading [1]. Furthermore, during a personalized cancer treatment various molecular markers coupled with specific antibodies allow the pattern of the growth of certain tumors and their response to the particular treatment to be predicted. For example, the proliferation marker Ki-67 is used in meningiomas to differentiate cancer into meningothelial (WHO I), atypical (WHO II), and anaplastic (WHO III) and correlates with tumor recurrences [1–5]. This is because the immunopositive signal expression is a surrogate measure of Ki-67 expression inside cells' nuclei. According to the World Health Organization (WHO) rules, the quantitative evaluation of the proliferation index is performed on a set of high power areas of hot spots selected in various places inside a whole specimen observed under a microscope. For each chosen area of selection, the number of immunopositive and immunonegative cell nuclei is counted to establish the Ki-67 index as the ratio of immunopositive cell nuclei to the whole number of cell nuclei. This routine practice lacks reproducibility from observer to observer because this definition is highly flexible. By definition, selected areas should represent fields of high Ki-67 index in different tumor localizations. The significant variability of possible selection leads to inter- and intraobserver variability in quantitative results which should be investigated in observer based assessment [6–16].

There have been many attempts to help histopathologists in Ki-67 index quantification involving computers and digital versions of the glass slide, called the whole slide image (WSI). A review of papers concerning this subject published both in the days when only small images could be handled by computers [17–24] and nowadays when WSIs are available and computers or clusters of computers have the necessary computing power to manipulate them [25–34] shows that investigators propose the use of computers on at least 3 levels of the process of proliferation index quantification: (1) in region selection, (2) in immunopositive and immunonegative cell nuclei selection, and (3) in proliferation and other index counting. While the third level is obvious and the second is widely explored, the first level is still poorly represented in the literature. There are methods of region selection concerning Hematoxylin and Eosin staining [35–37], while for Ki-67 stained with DAB and counterstained by Hematoxylin there are the studies published by Potts [34] and coworkers, Lu and coworkers [35], and Gavrielides and coworkers [7, 8]. The third group of investigators performed a pooling study and concluded that “… for validation study should be focused on specific pathology tasks to eliminate sources of variability that might dilute findings.” So, a validation study of a specific use of Digital Pathology, that is, in the quantification of the proliferation index based on Ki-67 used in meningiomas, is presented in this paper.

## 2. Materials and Methods

### 2.1. Glass Slide Preparation

The glass slides used in this study came from meningioma patients diagnosed or graded at the Department of Pathomorphology, the Military Institute of Medicine in Warsaw, Poland. They were divided into two sets of data according to two methods of preparation. In set A there were twenty-three glass slides (57%, 13 patients, in grade I; 30%, 7 patients, in grade II; and 13%, 3 patients, in grade III according to WHO scores) prepared from paraffin blocks which had been randomly chosen with respect to quality from the hospital archives. The Ki-67/MIB-1 immunohistochemical stained procedure was performed using a Dako Autostainer Link and the following chemical: FLEX Monoclonal Mouse Anti-Human Ki-67 Antigen Clone MIB-1 Ready-to-Use (Link) reference number IR626 from Dako. The staining was visualized using EnVision FLEX Target Retrieval Solution from Dako according to the procedure described in the user manual. All manual and mechanical activities were performed very carefully, because the samples were supposed to be model quality in comparison to the slides from set B.

In set B twenty-seven glass slides (70%, 19 patients, in grade I and 30%, 8 patients, in grade II) from routine hospital prognoses and grading using Ki-67/MIB were chosen to be involved in the study. All these slides had been prepared between 2011 and 2014 with or without Autostainer Link in a manual procedure using various chemicals purchased from Dako. Set B contained inhomogeneous WSI in terms of both the manner of preparation and the chemicals used. The overall quality of glass slides from set B was worse than that of glass slides from set A.

### 2.2. Microscope and Monitor Review of the Digitalized Glass Slides

The sets of glass slides were both scored by an experienced pathologist, henceforth known as expert, using an Olympus BX40 optical microscope with PlanApo objective. Then, the slides were digitalized using an Aperio ScanScope scanner for set A and a 3DHISTECH Panoramic II for set B. These were then reviewed on a calibrated EIZO FlexScan 22-inch monitor. The WSIs were acquired under 400x magnification with a resolution of 0.279 *µ*m and 0.38895 *µ*m per pixel for sets A and B, respectively. Digital images were reviewed using dedicated software prepared according to project requirements which allowed panning around with a mouse/trackball to view the WSI in various magnifications and to mark fields of quantification. This software was prepared in MATLAB using library Open Slide [38] to read WSI files.

To ensure comparability of an area examined by an expert under a microscope as one field of view and area of quantification chosen from digital WSI, the size of the rectangle which covered the same area as the microscopic circular field of view was determined. It was assumed that the microscopic field of view at 400x magnification represents around 0.12 mm^2^ of a tissue; the size of the digitized field of view was 1424 × 1064 pixels in set A and 1024 × 766 pixels in set B.

### 2.3. Observer Training and Environmental Adjustments

Two pathologists with 7 and 3 years of practice in meningioma sections quantification were asked to support this study. To minimize sources of variability, both observers were trained on the software they were to use and their environments were controlled: they used the same computer, monitor, and light in the room in order to eliminate environmental influences on the pathologists work.

The pathologists had an introductory session to become familiar with all the controls and interfaces which were necessary in the selection of hot spots and proper size areas for quantification by automatic software. Pathologists had been instructed the following:

- The interpretation of Ki-67 does not include the classification of the intensity of staining but the percentage of tumor cells with positive staining.
- They should find 20 areas of the size mentioned above with high populations of brown objects in comparison to the nearest neighborhoods, but these areas should be distributed among all hot spots which could be found in WSI.
- Each area should be at least 80% covered by tumor lesion and without any artifacts.

Cases where even one of pathologists was unable to score (because of a lack or inadequacy of the region of a hot spot) were removed from the analysis. During the area selection the leader of the project assisted the pathologists by offering hardware and software support but did not make any suggestions as to how to gather information about hot spots or how to choose areas for quantification.

### 2.4. Textural Features Applied in the Proposed Method

To find hot-spot localizations, a texture analysis was performed on WSI. The normalized probabilities ${\hat{P}}_{s}(i)$ and ${\hat{P}}_{d}(i)$ of the *i*th intensity on the basis of histograms of the sum and difference images [37] were used. These images were formed from the original image by applying the relative translation (*d*_1_, *d*_2_). Let *f*_*k*,*l*_ mean the intensity of a pixel at (*k*, *l*)th position in the gray scale (each of RGB channels) and the image was translated by a fixed displacement (*d*_1_, *d*_2_)

(1) sk,l=fk,l+fk+d1,l+d2,dk,l=fk,l−fk+d1,l+d2,

where *s* and *d* represent the sum and difference images. The normalized sum and difference probabilities were estimated by

(2) P^si=hsiN,P^di=hdiN,

where *N* is the total number of pixels in the image. We used the modified formulas of Unser features [37] which are presented in Table 1. They were applied over a given region *Ω* associated with each pixel of the image. In our notation, *N*_*Ω*_ represents the total number of pixels in Ω region, and *s*(**x**) and *d*(**x**) represent the pixel values of the sum and difference images.

The determination of the image resolution and Ω radius, which allow the best characterization of the local structures in images, was achieved.

The texture analysis was performed in the following steps. The sum and difference images on the basis of the original image and the original image translated by 3 pixels were calculated for each of the RGB channels. Then, the disk masks with a radius of 10 pixels selected the set of the neighborhood region masks for each pixel location. For a neighborhood of size of 5, 8, 10, 12, 15, and 20 pixels the radius size of 10 pixels appears to be the best and this was used in further experiments.

For the texture features defined in Table 1, the computation complexity problems were obvious. These were associated with the traveling location of the central pixel and its neighboring region Ω. This was solved by applying the array operations. The process of adding the pixel values in sum and difference images was realized quickly by applying the average filtering of the image (embedded* imfilter* function in MATLAB). Thereby, the mean mask for the whole image could be calculated in only one analysis. The (*k*, *l*)th coordinate of this mask represented the region center located in this point. To efficiently implement this method of feature calculation, the array form of operations was applied. For example, the variance feature (the second row in Table 1) could be computed according to the following (modified) expression:

(3) f2=∑x∈Ωsx2−4μΩ∑x∈Ωsx+4μΩ2NΩ+∑x∈Ωdx22NΩ.

The first term of this relation was calculated by applying the filtering of the array-squared sum image (the Hadamard product) and the second by array-fashion multiplication of the mean of the image and the filtered sum image. In the same way, the other terms were calculated. Thereby, the texture feature computation time has been significantly decreased.

### 2.5. Automatic Hot Spot and Area of Quantification Selection

The proposed method for the hot-spot localization and area of quantification selection based on mathematical morphology, texture classification, and controlled dispersion was described in this section.

**Figure 1:**
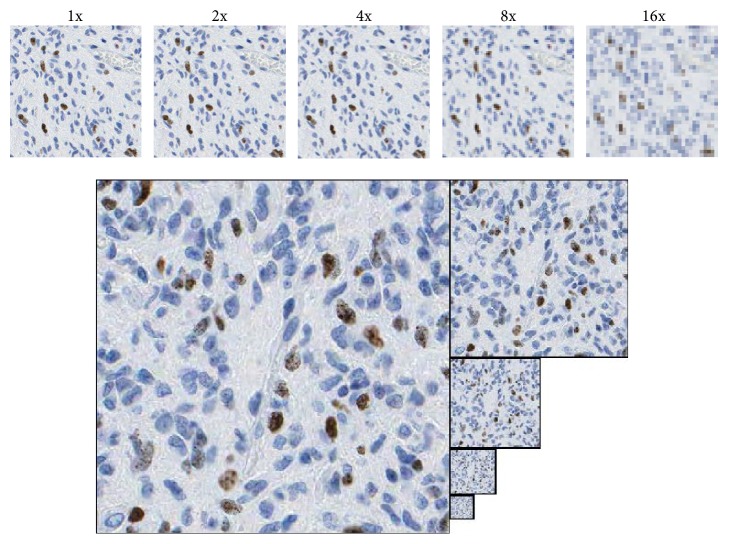
Fragments of WSI in original and decreased resolution: 2x, 4x, 8x, and 16x.

An analysis of the information contained in WSI after a resolution decrease on various scales showed that the texture in the original image is redundant and the resolution can be decreased. To localize hot spots, information about the ratio of brown (red) to blue pixels as a basic feature and some other features described below were needed. All features were also visible in images with the resolution decreased by up to 8x, while at a 16x decrease they were not visible. This is presented in Figure 1.

It appears that an eightfold reduction of the resolution does not disturb the required further textural features (size of object-cell nuclei is decreased from 128 ± 51 for brown and 102 ± 73 for blue in original image to 18 ± 9 and 10 ± 6 for the selected 8x decreased resolution, resp.) and enables the evaluation to be performed by a computer and by a pathologist with a direct visual examination.

**Figure 2:**
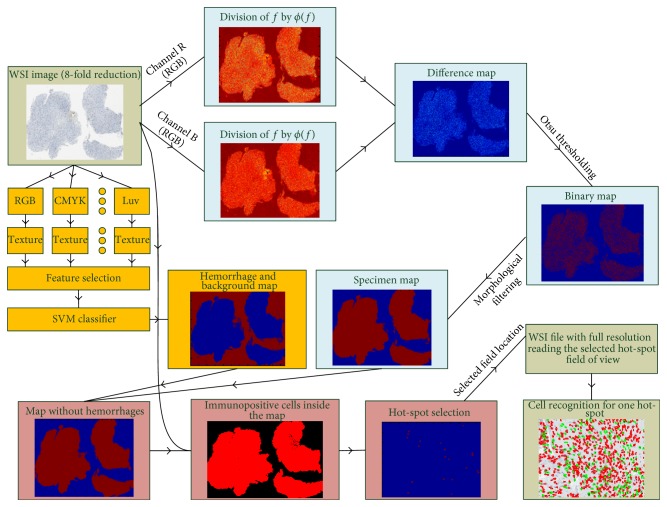
The schema of the algorithm for hot-spot localization.

The proposed method of analysis of WSI in decreased resolutions uses the following steps: (1) the specimen map is established, (2) the texture quantification and classification are done to eliminate hemorrhage areas from the specimen map, (3) the hot spots are detected, and finally, (4) based on the proposed penalty function, selection of the area of quantification inside selected hot spots is performed. The general schema of the algorithm for steps 1–3 is presented in Figure 2.

In the first step, a map of the specimen was created using the thresholding procedure and morphological filtering [39–42]. To do this, a whole slide image was used to produce a supported image by the morphological operation of opening and brightness equalization. This was performed using a structuring element shaped like a disk with a large radius (100 pixels). The operation of the division of each RGB color component of the image by its version after morphological opening was performed independently for each channel. Afterwards, components from channels B and R were processed with the Otsu thresholding method [43]. Additionally, morphological operations, such as erosion, dilatation, and hole filling, were performed to filter the specimen map.

The next step, which eliminates hemorrhage areas from the specimen map, was performed by differentiating the tumor area from hemorrhage areas using texture analysis and classification. The local textural descriptors came from the Unser features [33, 40] and were applied independently for RGB and CMYK color channels and also for the combined u (from CIE Luv) and C (form CMYK) representation. A set of 64 textures was created as 8 features defined in Table 1 by 8 color channels or sums of channels as presented in Table 2. Next, based on Fisher's linear discriminant, the most significant 25 were selected on a teaching phase and then used in the classification phases (see Table 2).

Finally, the Support Vector Machine (SVM) with Gaussian kernel function [41–46] was applied as a classifier to recognize the hemorrhage areas and to eliminate them from the specimen map.

The third step of the algorithm was an estimate of the local density of immunopositive cells using the reduced resolution WSI. The local maxima of the immunopositive cell densities are hot spots. To select these, the mathematical morphology and proportion of the color components were used. It was found that u of the CIE Luv representation of colors is strictly associated with the red color and can be used to differentiate the immunopositive cells from the remainder of the image. The extended regional minima transformation is applied to evaluate the spatial relation of the stained brown objects to their neighboring environment. The density map was created, based on the isolated marks representing the immunoreactive tumor cells.

The fourth and final step of the proposed method focused on the fields of quantification selection based on an artificial model of field spatial dispersion. To prevent all fields of quantification being chosen, the penalty function was defined from one large dominant hot spot with a high Ki-67 index by the following formula:

(4) $$\begin{array}{c} \text{penalty}=1-\rho \sum_{i}\frac{1}{{\left(\sqrt{{\left(x-x_{i}\right)}^{2}+{\left(y-y_{i}\right)}^{2}}\right)}^{0.5}} \end{array}$$

which was based on information about the distance between the designated areas and the position of another candidate for hot spot.

An increase in the *ρ* value shows an increase in the scattering of the areas of quantification selection. The *ρ* value had been chosen experimentally (see Section 3). The proposed function combined selection of fields of quantification from different localization in the specimen according to a gradual reduction of the concentration of immunopositive cells. However, when hot-spot areas other than the dominant one show a significantly lower density of immunopositive nuclei, the candidates from dominant region will still be selected first. The final analysis of the Ki-67 index in all chosen areas of quantification was performed on full resolution images with the method published earlier and described in [45].

### 2.6. Evaluation of the Concordance of Selected Hot-Spot Fields

To evaluate the concordance of hot-spot field localization between the experts' and automatic results the localization concordance measure (LCM) was proposed. This measure assumed that (1) those fields at a shorter distance should have a reduced impact on the LCM and (2) the significance of fields should relate to their Ki-67 index. The localization concordance measure was calculated according to the following formula:

(5) LCM=∑iwi∗sigm⁡minj⁡dist⁡xj,yj,xi,yi4FOVsize,where:  wi=LEiL−E

in which *L*_*E*_ is the level of the Ki-67 index for the expert and FOV_size_ is a one field of view size. A low value of the LCM shows a similarity in the areas of quantification selection by algorithm and expert. This means that the expert's selected fields of quantification are represented or near the fields selected by the proposed method; for example, they represent the same tumor area. If expert and algorithm select fields of view from different virtual slide areas, the localization measure LCM is high. The proposed measure allows both the evaluation of the similarity of choice of hot-spot fields and the identification of the best penalty factor. The proposed measure can be used in cases of both inter- and intraobservation variability.

### 2.7. Study Design

Both the proposed automated method of area of quantification selection and the two pathologists were used to review all the samples (A and B sets) using digital representation of glass slides with an 8x reduced WSI resolution while expert quantification was done in full resolution and for 10 areas of unknown location. The outcomes were then averaged to give the final result.

Each of the pathologists chose 20 fields of quantification for each WSI. One pathologist had two additional sessions for the WSI from set A to estimate interobserver variability. Each additional session was performed with at least a one-month delay between sessions. The order of samples was randomized for each session.

Then, automated hot-spot selection software was used to select 20 fields of quantification as an area which fulfill two criteria:

- Biggest number of immunopositive nuclei in comparison to the others.
- That distances between new and previously found areas are large enough to meet the requirements of the above defined penalty function, that is, (4).

The scores of the Ki-67 index from the areas of quantification chosen by the pathologists and the automatic method were produced using software which segments nuclei in subimages from WSI in full resolution and then classifies them into immunopositive and immunonegative classes and estimates the Ki-67 index. This software was published in 2009 [45] by the principal investigator of the project who is coauthor of this paper. The ratio of immunopositive nuclei to the number of all cells in each area of quantification and the mean of these ratios for each WSI were sent for computer-enabled statistical analysis.

### 2.8. Statistical Analysis

The scores from the expert microscopic examination and all automatic scores from areas chosen by both pathologists and scores from the proposed automatic method of area of quantification selection were analyzed using agreement analysis, since IHC interpretation is a subjective process of evaluation. For this process, a true score is not available. Besides, agreement between digital and optical scores was not the primary objective of the study, but rather this is considered the reference value, while agreement between the automatic and pathologists' hot spots and area of quantification selection in IHC assessment was the main aim of the study.

The primary objective of the investigation was to find patterns of agreement between manual human and automated selection of the area of quantification in WSI. The commonly used concordance measure, Kendall's tau-b, was used as in Gavrielides and coworkers [7, 8]. The test was calculated separately for sets A and B in pooled and categorized/grouped versions in both pairwise and cumulated versions.

Kendall's tau-b is a rank-based correlation metric which calculates the difference between the rate of concordance and discordance [46–48]. The range of Kendall's tau-b is −1 to 1, where 1 indicates perfect agreement, −1 indicates data are inverted (perfect agreement inversion), and 0 indicates no relationship. Kendall's tau-b was computed according to Balboacă and Jäntschi [48] using dedicated software prepared in MATLAB.

Kendall's tau-b values were utilized to quantify* interobserver *and* intraobserver agreements. *The* interobserver agreement* was estimated between all pairs: (1) between pathologists themselves, ((2) and (3)) between each pathologist and classical expert microscopic reviewing, ((4) and (5)) between each pathologist and the proposed automated method and additionally in a grouped version between (6) the mean of pathologist and classical expert microscopic reviewing and (7) the mean of the pathologist and proposed automated method applied to WSI and (8) between classical expert microscopic reviewing and proposed automated method applied to WSI. The* intraobserver agreement* (agreement between the scores of the same observer in various sessions of area selection) was estimated between all pairs of three independent scorings from one pathologist: (1) the first and the second scores, (2) the second and the third scores, and (3) the first and the third scores, both in pooled data and in categorized data. Because of the small number of WSIs from patients in grade III, the results relate to only two categories: grade I and grade II in diagnosed meningioma patients. Confidence intervals for the overall agreement measures were calculated applying bootstrap analysis using a procedure described in detail in the study by Gavrielides et al. [7].

Software for bootstrap was implemented using MATLAB (MathWorks, Natick, MA, USA) functions.

## 3. Results

**Figure 3:**
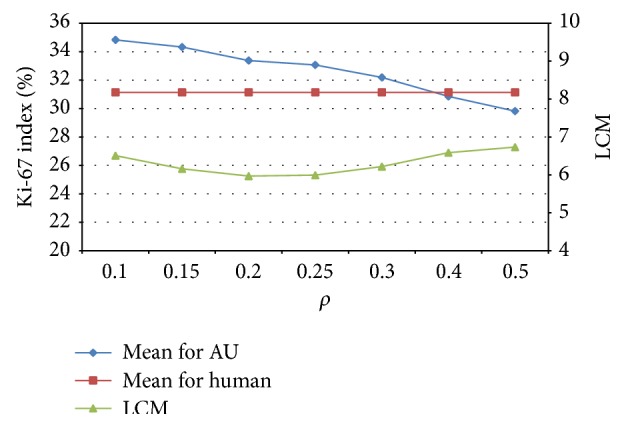
Examination of Ki-67 index and LCM in respect to the *ρ* factor of the penalty function.

First, the influence of the *ρ* value on the penalty factor was examined. A subset of twelve WSIs from set A was chosen for this analysis. The hot-spot localization and areas of quantification selections performed by pathologists and the automated proposed method were compared using the LCM measure. The results of Ki-67 index estimations and LCM measures for *ρ* from 0.1 to 0.5 with an increment of 0.05 are presented in Figure 3. The best concordance between the automatically selected areas of quantification and those selected by pathologists is for a *ρ* value equal to 0.2. For this *ρ* value, LCM is minimal for a relatively high value of the Ki-67 index.

**Figure 4:**
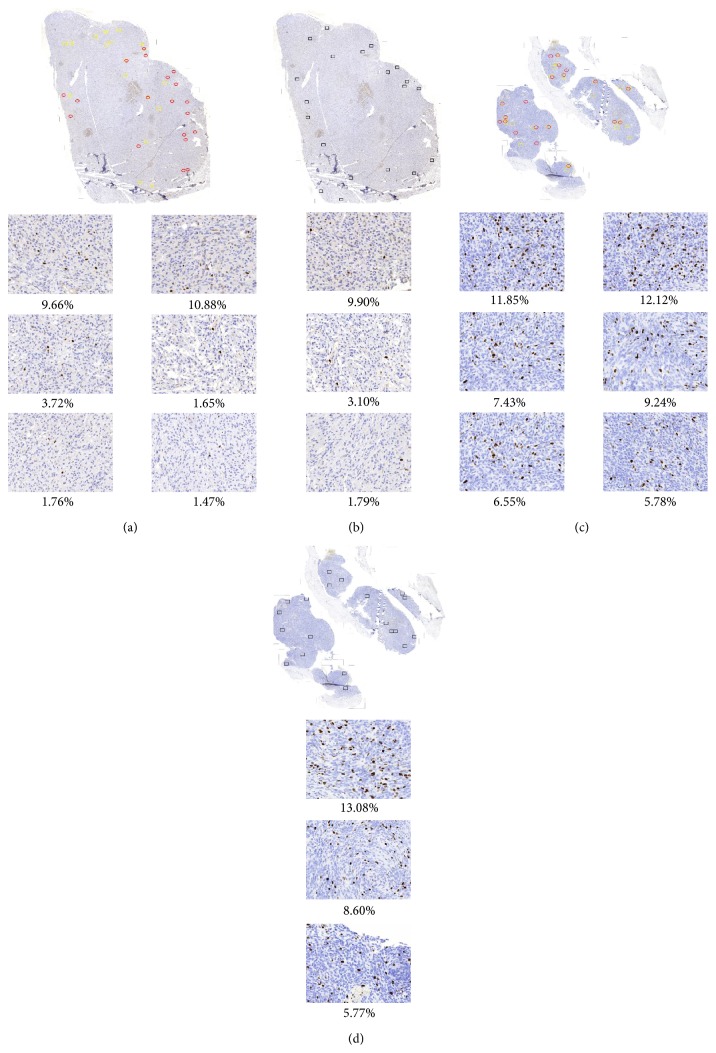
Two WSIs with an area of quantification inside a hot spot marked by two experts ((a) and (c): red and yellow rectangles) and chosen by the proposed automatic method ((b) and (d): black rectangles) with examples of 3 areas of quantification for each method and for both WSIs with large and small variation in Ki-67 index value calculated as percentage of immunopositive nuclei to the whole number of nuclei in presented area.

**Figure 5:**
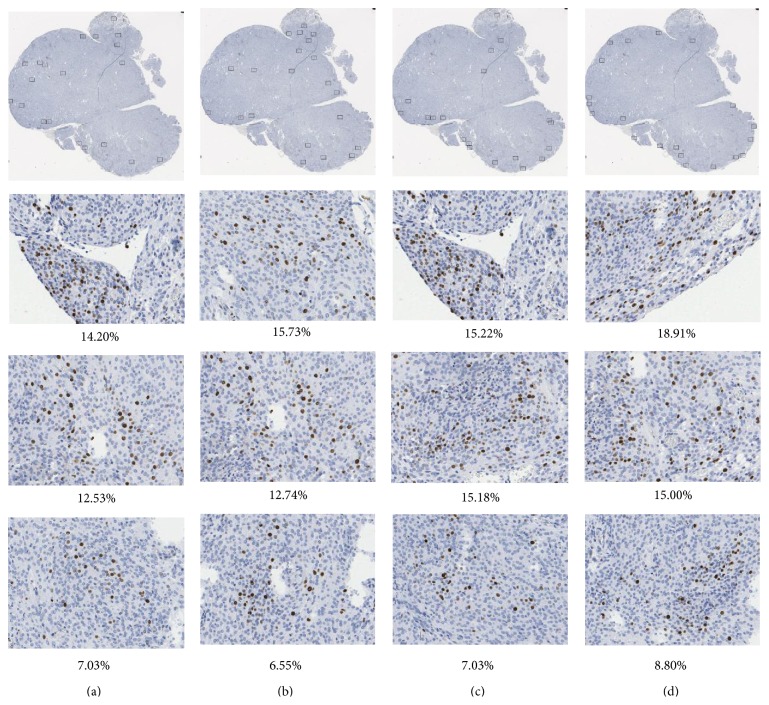
The results of hot spot and area of quantification selections by one pathologist ((a), (b), and (c)) and by proposed automated method (d) with examples of 3 areas of quantification chosen approximately in the same region (hot spot) for each selection. The Ki-67 index value calculated as percentage of immunopositive nuclei to the whole number of nuclei in presented area differs slightly.

The dispersion of areas of quantification chosen by the proposed automatic method and pathologists can be observed in Figures 4 and 5.

Figure 4 presents the distribution of the areas of quantification selected in hot spots found for two WSIs by two pathologists (red and yellow rectangles) and by the proposed automatic method (black rectangles). In the top line (Figures 4(a) and 4(b)) it can be observed that there is no agreement between both pathologists and that the distribution of regions is different and inhomogeneous, so the measure of concordance, LCM, is 9. The distance between the proposed automatic method and the mean measure for both pathologists is 8.6. The bottom line (Figures 4(c) and 4(d)) shows good agreement in areas of quantification distribution. Their measure of concordance, LCM, is 2.9 between pathologists while between the proposed automatic method and the mean measure for both pathologists it is 3.4.

Figure 5 presents results of three repetitions of the selections of the areas of quantification from one pathologist (Figures 5(a), 5(b), and 5(c), blind trial) and from automated method (Figure 5(d)) using one of the WSIs from set A. It is visible that this one person chose a region of quantification in various parts of specimen. The third trial is significantly different from the previous two rather similar trials, but the Ki-67 indexes for each of them are similar (10.7%, 10.6%, and 11.9% for pathologist and 13.2% for automated method).

As the selected fields of quantification were previously quantified by the software, the Ki-67 index for each area and for each specimen becomes the data for statistical analysis.

**Figure 6:**
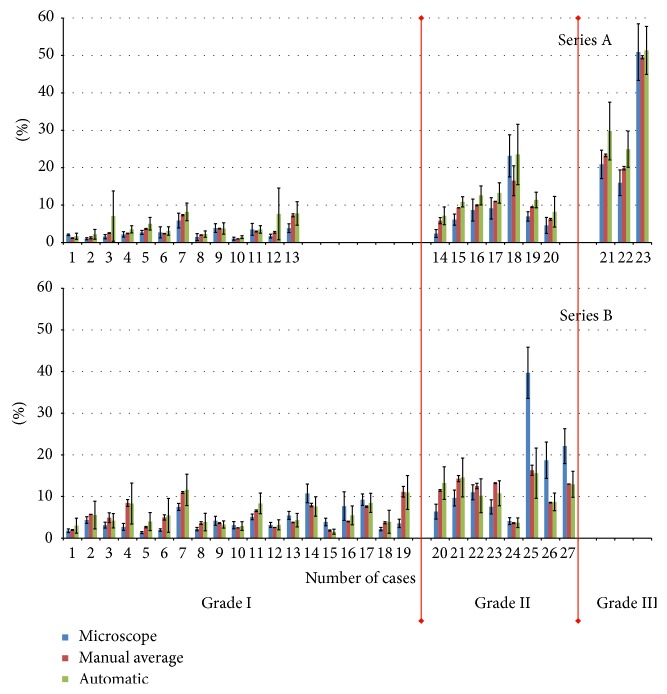
The mean and standard deviation of the scoring Ki-67 index for all 50 examined glass slides and their WSI derived from meningioma patients. Data are grouped according to the types of measurements, grade of malignance (grade I, grade II, and grade III), and type of sample preparation method (sets A and B). In each of the three bars the first, blue bar shows the result for the manual microscopic expert score and the second, red bar shows the result for the mean of the pathologists' quantifications using WSI, while the third, green bar presents results for the proposed automated method.

In Figure 6, where all results for Ki-67 quantification using all early interdicted methods of its estimation (manually experts, by means of two pathologists' semiautomatic approach and fully automatic approach) are shown, the general tendency for a relationship between them can be observed. Quantification with the manual microscopic method produces the lowest Ki-67 proliferation index values, while the automatic methods produce the highest values of this index. This pattern is biased by one specimen from set A and for 3 specimens from set B. In the first case, the lower result for the pathologists is caused by an undervalued score from one pathologist. In the other cases from set B, the pattern is reversed and the highest values for this index appear for the manual microscopic method. Exposé control of WSI shows that there are very small hot spots in each of these three specimens. It seems that when hot spots do not cover the whole area of quantification (although they fulfill the criterion that about 80% of the area of quantification should be covered by a hot spot) it causes various results from the pathologists and automatic method. In such cases, an expert performing microscopic scoring used to deal with part of field of quantification restricted to hot spots, while the automatic method diluted the score by counting the number of cells from the whole area of the rectangle.

The results of inter- and intraobserver variability measurements are presented in Tables 3, 4, and 5.

Table 3 presents the results of pairwise agreement using Kendall's tau-b analysis for interobserver variability as a coefficient of concordance along with confidence intervals (95% confidence level) constructed using bootstrap analysis of the samples (100 order changes). It can be observed that all concordance between all pairs calculated for set A is bigger than the analogous values for set B. This can be explained by the fact that WSIs in set A were prepared using autostainer and the same set of new chemicals while WSIs in set B were regular glass slides prepared earlier, some with and some without autostainer, and using the chemicals available at the time. Visual examination shows that glasses in set A are of really good quality and homogenous in performance, while the glasses from set B are not. This inhomogeneity among glasses from set B led to differing interpretations by the two pathologists which is seen as a decrease in agreement between them (from 0.92 to 0.86) and between each of them and the automated method (from 0.82 and 0.81 to 0.78 and 0.76, resp.).

Both parts of Table 3, A and B, show an overall tendency for the highest correlation to be between both pathologists and the lowest agreement to be between a classical microscopic expert scoring and the proposed automated method employed on WSI. The concordance between both pathologists and the other two methods of scoring are between these two extremes, but the concordance is greater between the pathologist and the proposed automated method than between the pathologists and the classical microscopic score.

Table 4 presents the results of pooled expert agreement in two categories, grade I and grade II (WHO categorization of meningioma), using Kendall's tau-b analysis for interobserver variability. The coefficient of concordance is presented with confidence intervals (95% confidence level) calculated using bootstrap analysis of the samples (100 order changes). It can be observed that concordance is higher in category grade II than in grade I in both subsets: A and B. This fact can be explained as the reason that grade I patients' scores are usually lower (up to 8%; see Figure 4) than grade II patients' scores (up to 20%; see Figure 4). This means that the hot spots are more intensive and visible in comparison to the surrounding space in those specimens from patients diagnosed as grade II. This visibility is more important for the pathologists than for the automated method. So, coincidence between pathologists' digital scoring and the proposed automated method is very high (0.86 for set A and 0.8 for set B) if grade II patients' sections are analyzed, while for grade I patients' sections the coincidence is lower (0.79 and 0.78), but it still shows coincidence. For the coincidence between experts' digital scoring and the manual microscopic expert scoring the results show similar patterns, but the numbers are lower (0.87, 0.43 and 0.6, and 0.3, resp.).

The coincidence between the results of the proposed automatic method and manual microscopic expert scoring is ambiguous. This coincidence is rather low, except for grade II of set A.

Table 5 presents the results of pairwise agreement analysis for uncategorized and categorized data using Kendall's tau-b for intraobserver variability. One of the pathologists repeated the scoring procedure 3 times, with a delay long enough to forget the samples. The results presented coefficients of concordance supported with confidence intervals (95% confidence level) calculated using bootstrap analysis of the samples (100 order changes). This shows very good agreement for all combinations of three scores performed by one observer (all coefficients are between 0.85 and 0.9 for data without categorization and between 0.7 and 0.85 for those categorized in grade I and between 0.73 and 1 for those categorized in grade II). Intraobserver variability is significantly smaller than analogous interobserver analysis results presented in Table 4.

## 4. Discussion and Conclusions

**Figure 7:**
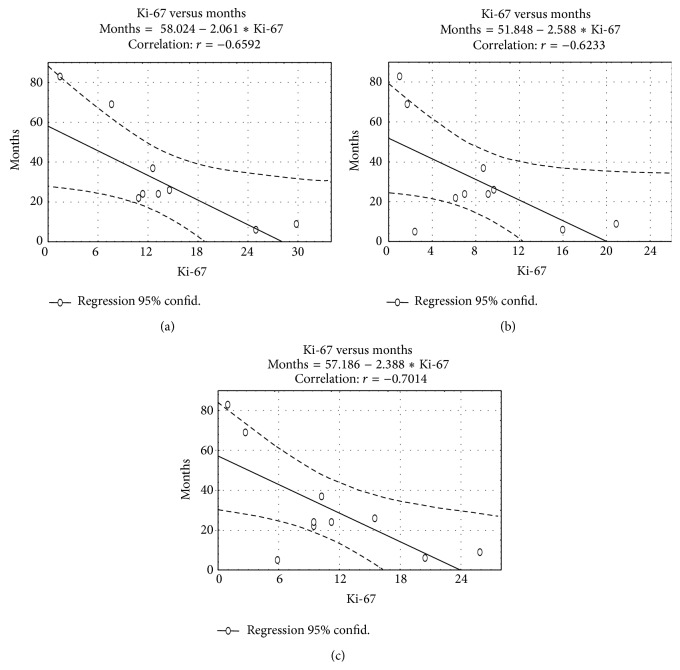
The regression function estimated for the number of months between recurrences of meningioma related to the value of Ki-67 index calculated based on (i) fully automatic method (a), (ii) traditional microscopic assessment (b), and (iii) semiautomatic human-computer hybrid approach (c).

The Ki-67 index, obtained for each patient WSI, determines the downstream clinical decision which concerns patients' treatment and, in consequence, patients' recovery, recurrences of the disease, or patient death. To compare the Ki-67 index obtained using three methods, that is, traditional microscopic, human-computer hybrid method, and the fully automatic method proposed in this paper in the context of the final results of the therapy for the patients, there is a need to know full patients' case histories which are not available in the Polish Healthcare System. What appears to be available after exposé documentation review is data on the recurrence of meningioma in those patients who have been rehospitalized in the same hospital. Among 50 patients whose samples or glasses were used in these investigations, only 10 patients have currently returned to the same hospital with a recurrence of meningioma. So, the prediction of the probability of the meningioma recurrence based on the Ki-67 index for all three methods of estimation has been estimated. The regression functions calculated for the number of months between the cancer surgical treatment and its recurrence in relation to the value of the Ki-67 index calculated based on 10 patients' information are presented in Figure 7.

Comparing all three regression function parameters (*ax* + *b*) and the value of the correlation, there is no significant difference between them.

In summary, the results of both the above analysis and the analysis described in the previous section show that there is no evidence that either hybrid human-computer aided or fully automatic selection of the area of quantification is superior for quantifying the Ki-67 index in meningioma patient samples. The results of the study show close agreement in terms of their correlations with tumor recurrences and a relatively high overall agreement for quantification using both methods presented in the paper, while the results for each of the methods and traditional macroscopic estimation by an expert are not so high.

In this study, the time constraints were not examined but without any doubt the automatic area selection followed by automatic analyses would lead to time saving for pathologists.

The agreement observed for the three scoring methods, that is, traditional optical microscope and the method based on digital modalities used by pathologists to select the region of quantification, together with a fully automatic computer aided version of this selection, shows that automation of area selection in WSI is an effective tool in helping physicians and in increasing the reliability of diagnosis based on immunohistochemically stained tissue sections. Furthermore, discussion of the standardization of meningioma Ki-67 quantification is welcomed.

## Figures and Tables

**Table 1:** Modified definitions of Unser features.

Name	Modified computational formula
Mean	$f_{1}=\frac{\sum_{x\in \Omega }s\left(x\right)}{2N_{\Omega }}=\mu_{\Omega }$

Variance	$f_{2}=\frac{1}{2}\left(\frac{\sum_{x\in \Omega }{\left(s\left(x\right)-2\mu_{\Omega }\right)}^{2}+\sum_{x\in \Omega }d{\left(x\right)}^{2}}{N_{\Omega }}\right)$

Energy	$f_{3}=\frac{\sum_{x\in \Omega }s{\left(x\right)}^{2}\cdot \sum_{x\in \Omega }d{\left(x\right)}^{2}}{N_{\Omega }^{2}}$

Correlation	$f_{4}=\frac{1}{2}\left(\frac{\sum_{x\in \Omega }{\left(s\left(x\right)-2\cdot \mu_{\Omega }\right)}^{2}-\sum_{x\in \Omega }d{\left(x\right)}^{2}}{N_{\Omega }}\right)$

Contrast	$f_{5}=\frac{\sum_{x\in \Omega }d{\left(x\right)}^{2}}{N_{\Omega }}$

Homogeneity	$f_{6}=\frac{\sum_{x\in \Omega }\left(1/1+d{\left(x\right)}^{2}\right)}{N_{\Omega }}$

Cluster shade	$f_{7}=\frac{\sum_{x\in \Omega }{\left(s\left(x\right)-2\mu_{\Omega }\right)}^{3}}{N_{\Omega }}$

Cluster prominence	$f_{8}=\frac{\sum_{x\in \Omega }{\left(s\left(x\right)-2\mu_{\Omega }\right)}^{4}}{N_{\Omega }}$

**(a):** 

Color component	R (1–8)	G (9–16)	B (17–24)	u (luv) + C (CMYK) (25–32)	C (33–40)	M (41–48)	Y (49–56)	K (57–64)

Number of features	3, 5	11, 13	—	25, 26, 27, 29, 30, 32	33, 34, 35, 37, 38, 40	41, 42, 43, 45, 46, 48	49, 54	63

Feature name	(i) Energy (ii) Contrast	(i) Energy (ii) Contrast		(i) PSM (ii) Variance (iii) Energy (iv) Contrast (v) Homogeneity (vi) Cluster prominence	(i) PSM (ii) Variance (iii) Energy (iv) Contrast (v) Homogeneity (vi) Cluster prominence	(i) PSM (ii) Variance (iii) Energy (iv) Contrast (v) Homogeneity (vi) Cluster prominence	(i) PSM (ii) Homogeneity	Cluster shade

**(b):** 

1	2	3	4	5	6	7	8
PSM	Variance	Energy	Correlation	Contrast	Homogeneity	Cluster shade	Cluster prominence
(u + C), C, M, Y	(u + C), C, M	R, G, (u + C), C, M	—	R, G, (u + C), C, M	(u + C), C, M, Y	K	(u + C), C, M

**Table 3:** The results of the pairwise agreement analysis of data, without taking into account categorical information produced using Kendall's tau-b analysis for interobserver variability on the WSI from sets A and B separately: TM means classical expert microscopic review, P1 and P2 mean pathologists, and AU means proposed automatic method.

	TM	P1	P2	AU
A				
TM		0.85281 (0.78355 : 0.87879)	0.79221 (0.71429 : 0.82684)	0.68831 (0.60173 : 0.68831)
P1	0.85281 (0.78355 : 0.87619)		0.92208 (0.88745 : 0.93074)	0.81818 (0.76623 : 0.81818)
P2	0.79221 (0.71429 : 0.82684)	0.92208 (0.88745 : 0.93074)		0.80952 (0.75758 : 0.80952)
AU	0.68831 (0.60173 : 0.68831)	0.81818 (0.76623 : 0.81818)	0.80952 (0.75758 : 0.80952)	

B				
TM		0.67687 (0.65 : 0.70017)	0.58503 (0.55629 : 0.60697)	0.54762 (0.51888 : 0.56871)
P1	0.67687 (0.64966 : 0.70034)		0.86735 (0.85782 : 0.87398)	0.78231 (0.77262 : 0.78912)
P2	0.58503 (0.55629 : 0.60714)	0.86735 (0.85748 : 0.87381)		0.7551 (0.75 : 0.76531)
AU	0.54762 (0.51871 : 0.56871)	0.78231 (0.77228 : 0.78912)	0.7551 (0.75 : 0.76531)	

**Table 4:** The results of pooled (for all experts) agreement analysis of categorized data using Kendall's tau-b analysis for interobserver variability on the WSI from sets A and B separately. The data are grouped in categories, grade I and grade II, while grade III was excluded from the analysis because of the small number of WSIs (3) in this category. TM means classical expert microscopic review, Human means the mean of the pathologists, and AU means the proposed automatic method.

	Human	TM	AU
A—grade I			
Human		0.60606 (0.54545 : 0.69697)	0.78788 (0.66667 : 0.78788)
TM	0.60606 (0.54545 : 0.69697)		0.39394 (0.27273 : 0.42424)
AU	0.78788 (0.66667 : 0.78788)	0.39394 (0.27273 : 0.42424)	

A—grade II			
Human		0.86667 (0.46667 : 0.86667)	0.86667 (0.46667 : 0.86667)
TM	0.86667 (0.46667 : 0.86667)		1 (0.46667 : 1)
AU	0.86667 (0.46667 : 0.86667)	1 (0.46667 : 1)	

B—grade I			
Human		0.33333 (0.28105 : 0.39608)	0.77778 (0.75163 : 0.80392)
TM	0.33333 (0.28105 : 0.39869)		0.32026 (0.26797 : 0.35948)
AU	0.77778 (0.75163 : 0.80392)	0.32026 (0.26928 : 0.35948)	

B—grade II			
Human		0.42857 (0.2381 : 0.52381)	0.80952 (0.71429 : 0.90476)
TM	0.42857 (0.2381 : 0.52381)		0.2381 (0.047619 : 0.33333)
AU	0.80952 (0.71429 : 0.90476)	0.2381 (0.047619 : 0.33333)	

**Table 5:** The results of intraobserver agreement examinations using Kendall's tau-b analysis calculated based on pairwise analysis performed in uncategorized (top part) and categorized schemas. The results show coincidence between Ki-67 indexes based on 3 selections of fields of quantification by the same pathologist.

	P1 (1)	P1 (2)	P1 (3)
All			
P1 (1)		0.85004 (0.8355 : 0.88658)	0.9032 (0.88398 : 0.91775)
P1 (2)	0.85948 (0.8329 : 0.88745)		0.88216 (0.85541 : 0.90649)
P1 (3)	0.86165 (0.83203 : 0.8961)	0.89835 (0.87965 : 0.92554)	

Grade I			
P1 (1)		0.69697 (0.66667 : 0.72727)	0.84848 (0.72727 : 0.87879)
P1 (2)	0.69697 (0.66667 : 0.72727)		0.78788 (0.75758 : 0.84848)
P1 (3)	0.84848 (0.72727 : 0.87879)	0.78788 (0.75758 : 0.84848)	

Grade II			
P1 (1)		0.73333 (0.73333 : 0.73333)	0.73333 (0.73333 : 0.73333)
P1 (2)	0.73333 (0.73333 : 0.73333)		1 (0.86667 : 1)
P1 (3)	0.73333 (0.73333 : 0.73333)	1 (0.86667 : 1)	
